# Only child syndrome in snakes: Eggs incubated alone produce asocial individuals

**DOI:** 10.1038/srep35752

**Published:** 2016-10-20

**Authors:** Fabien Aubret, Florent Bignon, Philippe J. R. Kok, Gaëlle Blanvillain

**Affiliations:** 1 Station d’Ecologie Théorique et Expérimentale, CNRS, UMR 5321, 09200 Moulis, France; 2 Amphibian Evolution Lab, Biology Department, Vrije Universiteit Brussel, 2 Pleinlaan, B-1050 Brussels, Belgium

## Abstract

Egg-clustering and communal nesting behaviours provide advantages to offspring. Advantages range from anti-predatory benefits, maintenance of moisture and temperature levels within the nest, preventing the eggs from rolling, to enabling hatching synchrony through embryo communication. It was recently suggested that embryo communication may extend beyond development fine-tuning, and potentially convey information about the quality of the natal environment as well as provide an indication of forthcoming competition amongst siblings, conspecifics or even heterospecifics. Here we show that preventing embryos from communicating not only altered development rates but also strongly influenced post-natal social behaviour in snakes. Clutches of water snakes, *Natrix maura*, were split evenly into half-clutches and incubated as (1) clusters (i.e. eggs in physical contact with each other) or (2) as single eggs placed in individual goblets (i.e. no physical contact amongst sibling eggs). Single incubated eggs produced less-sociable young snakes than their siblings that were incubated in a cluster: the former were more active, less aggregated and physically contacted each other less often than the latter. Potential long-term effects and evolutionary drivers for this new example of informed dispersal are discussed.

Egg-clustering and communal nesting behaviours are traditionally assumed to have evolved as anti-predatory tactics; from extinct dinosaurs^1^ to insects^2^, spiders^3^, cephalopods^4^, fish^5^ to amphibians^6^. Yet, recent studies challenged this view by reporting direct benefits of egg clustering and communal nesting to offspring phenotypes: hydric modifications of incubation conditions^7^, heating via the metabolic output of neighbouring eggs^8^,^9^,^10^,^11^,^12^, securing and maintaining egg/embryo positioning throughout the incubation^13^, and by enabling embryo communication amongst developing eggs within or amongst clutches^14^,^15^,^16^.

Embryo communication was recently discovered in both avian and non**-**avian reptile clutches^17^,^18^,^19^,^20^. Cues such as sound production, egg vibration, heart rates, odours, or carbon dioxide levels within the nest were proposed as potential communication avenues amongst embryos^17^. Embryo communication was shown to primarily promote hatching synchrony via metabolic compensation between more and less advanced eggs^16^,^17^,^18^,^21^ or between large and smaller eggs^22^. Synchronised hatching is wide-spread amongst organisms, including squamates (snakes and lizards), and is thought to enhance offspring survival by diluting an individual’s risk of predation or by simply swamping predators upon emergence^23^,^24^,^25^,^26^. Yet it was recently hypothesised that the kind of information exchanged between developing embryos may extend beyond metabolic rates, and include stress, sex ratio, relatedness or clutch size and therefore potentially convey information about the quality of the natal environment as well as an estimate of forthcoming competition amongst siblings, conspecifics or even heterospecifics^14^,^16^. As such, the long-term effects of embryo communication (personality, dispersal, survival) constitute novel and intriguing research avenues at an overlooked life-stage.

We experimentally manipulated clutches of water snakes (*Natrix maura*) to allow or prevent communication between embryos. Clutches were split evenly into half-clutches incubated as (1) clusters (i.e. eggs in physical contact with each other) or (2) as single eggs placed in individual goblets (i.e. no physical contact amongst sibling eggs). We recorded embryo development rates (heart rate trajectories), hatchling body size and post-natal social behaviour in young snakes.

## Results

### Incubation and hatching traits

**Figure 1:**
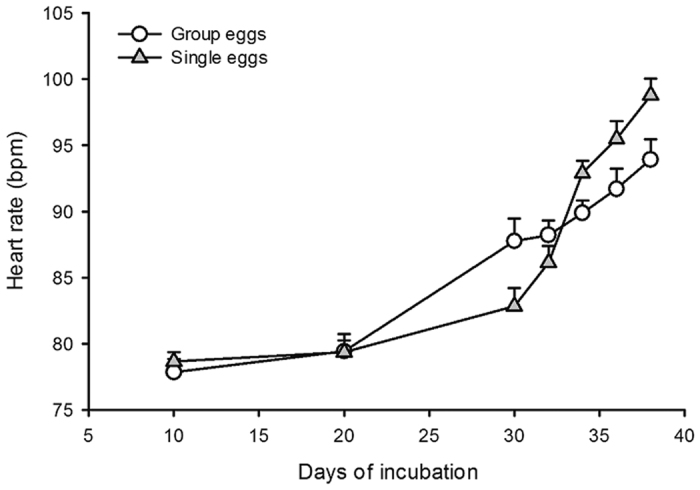
Five clutches of water snakes *Natrix maura* were split evenly into half-clutches incubated as (1) clusters (i.e. eggs in physical contact with each other: N = 28 eggs) or (2) as single eggs placed in individual goblets (i.e. no physical contact amongst sibling eggs; average half-clutch size = 5.60 ± 1.95 eggs; N = 28 eggs) in order to respectively allow or prevent communication between embryos. Embryo heart rates were monitored for each egg throughout the incubation period. Heart rate trajectories were significantly altered by the treatment (group; open circles versus single eggs; grey triangle): repeated measure two factors Anova: interaction term F_24, 270_ = 1.75, P < 0.019; effect of treatment on heart rates over time F_6, 270_ = 7.89, P < 0.0001. Means ± SE are plotted.

Egg mass did not significantly vary between treatments throughout the incubation period (Table 1). There was however a significant effect of treatment on heart rate trajectories throughout the incubation period (see Table 2 and Fig. 1). The alteration of heart rates trajectories likely related to altered metabolic rates and utilisation of yolk (unabsorbed yolk was found in larger quantities in single eggs after hatching; Table 3). Yet the effects on hatchling body size and incubation time were non-significant (Table 3).

### Aggregation and activity

**Figure 2:**
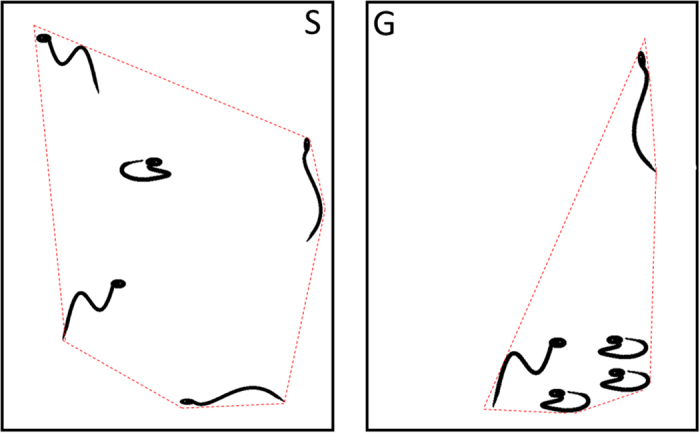
The social behaviour of young sibling *Natrix maura* was recorded in open boxes using a digital video camera fitted above the testing arenas. Snakes were either born from eggs incubated in individual goblets (“single”, box S) or as a cluster (“group”, box G). Every two minutes for a two-hour period we calculated the area occupied by the 5 snakes (dotted red line) using snapshots from the video. Areas were calculated using open source software ImageJ.

**Figure 3:**
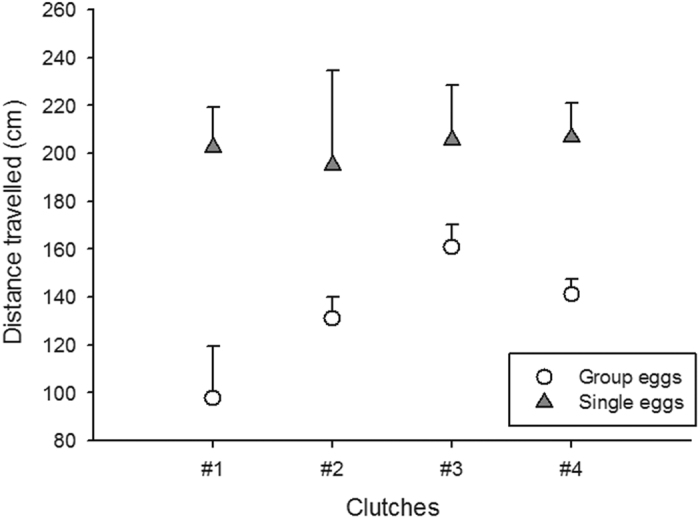
The distance travelled by each snake was recorded over one minute every 15 minutes of the two hours long video recording using Photofiltre 7 with the plugin “mesures”. Individual snakes incubated alone (grey triangles) covered on average 202.47 ± 11.67 *versus* 132.59 ± 8.01 cm for their siblings incubated as a clutch (open circles). The effect was consistent across clutches (two factor Anova; interaction term F_3, 32_ = 0.78, P = 0.51; effect of treatment F_1, 32_ = 23.96, P < 0.0001). Means ± SE are plotted.

The area occupied by the groups of 5 snakes strongly differed between treatments (repeated measure Anova; interaction term F_60, 360_ = 0.89, P = 0.71; effect of time F_60, 360_ = 0.96, P = 0.55; effect of treatment F_1, 6_ = 17.13, P < 0.0061; Fig. 2). Siblings incubated alone occupied an average area of 695.48 ± 31.67 cm^2^ *versus* 433.31 ± 122.65 cm^2^ for siblings incubated as a group. Individual snakes incubated alone were also more active and covered more distance on average than their siblings incubated as a clutch (two factor Anova; interaction term F_3, 32_ = 0.78, P = 0.51; effect of treatment F_1, 32_ = 23.96, P < 0.0001; Fig. 3). Finally, snakes incubated alone physically contacted each other less (N = 5.90 ± 1.80 contacts) than snakes incubated as a clutch (N = 6.95 ± 2.09; two factor Anova, interaction term F_3, 32_ = 2.26, P = 0.11; effect of clutch of origin F_3, 32_ = 5.81, P < 0.0028; effect of treatment F_1, 32_ = 4.28, P < 0.047).

## Discussion

This experiment showed that single *versus* group incubation altered developmental rates (heart rate trajectories and yolk utilisation were altered) in developing snake embryos. Hatchling size was also affected but the difference between treatments fell short of statistical significance, perhaps due to relatively low sample sizes. These results are broadly consistent with earlier studies where skink^7^ and turtle^21^ eggs incubated within a cluster produced hatchlings that were larger than hatchlings from eggs incubated alone.

The treatment on the other hand strongly modified post hatching behaviour, in a somehow counterintuitive manner: single eggs produced less-sociable young snakes than eggs than had been incubated as a cluster. While it was assumed that communal nesting/clustering in egg laying animals offered a range of advantages^7^,^8^,^9^,^10^,^11^,^12^,^13^,^14^,^15^,^16^,^17^ (from anti-predatory benefits, maintenance of hydric balance, maintenance of egg positioning to hatching synchrony), this study suggests yet another driver for the evolution and maintenance of these reproductive traits. Egg clustering, and incidentally embryo communication^14^,^15^,^16^, may foster the establishment of “social bonds” amongst embryos. The experimental rupture of this alleged bond not only modified developmental rates but also altered post-natal activity levels and aggregative patterns amongst siblings. This possibility had been suggested previously^27^: communal oviposition, by generating intra**-**clutch (and sometimes inter**-**clutch) hatching synchrony in squamates, may allow social interaction to occur. For instance, synchronously hatched *Anolis carolinensis*^28^,^29^ and *Sceloporus jarrovi*^30^ are known to exhibit social displays soon after birth. Neonate snakes *Storeria dekayi*^31^, *Thamnophis sirtalis*^31^, *Nerodia sipedon*^32^, *Crotalus horridus*^33^, and *Crotalus viridis*^34^ were shown to be attracted to neonate conspecifics or associated chemical cues. In *Iguana iguana*, hatchlings emerge and disperse from communal oviposition sites synchronously^35^, while engaging in social behaviours^36^,^37^,^38^. It was shown that groups of hatchling iguanas tend to disperse in the “correct” direction (i.e., toward the shortest route to the mainland) more frequently than single hatchlings^39^. Our study on water snakes support these findings but further suggests that these social bonds may originate not only from immediate interaction following synchronous hatching and odour imprinting, but from well before hatching as a result of embryo to embryo communication within the nest, possibly mediated by heart beats^16^ and/or hormonal or odours clues.

Future studies may address the evolutionary nature of these results by (1) demonstrating the occurrence of similar patterns in other taxa, (2) describe communication mechanisms and (3) assess the long term effects of single *versus* grouped incubation on dispersal behaviour. Yet we can speculate that the creation and maintenance of social bonds amongst communally laid eggs may provide anti-predatory benefits by fostering aggregative behaviour, thereby diluting the risks of predation^23^,^24^,^25^,^26^ and providing thermal benefits to the offspring^40^; or generating more efficient dispersal^39^. Alternatively, embryo communication may allow developing embryos to assess the number of siblings and /or non-related conspecifics within a clutch or communal nesting site. It is plausible that few or no neighbouring eggs may be indicative of high predation levels on the eggs, or alternatively of a resource poor environment (i.e. low reproductive output) from which rapid dispersal is required. On the other hand, a larger clutch (i.e. numerous neighbouring egg) may be indicative of relatively safer and/or resource full environment from which there is no direct advantage to disperse from, given that dispersal is risky^41^. The latter suggests a new form of informed dispersal^42^,^43^. Further studies may test whether these non-exclusive and fascinating possibilities may explain single-child syndromes in snakes in particular and egg-laying animals in general.

## Methods

Gravid female *Natrix maura* were captured in June and July 2014 along the banks of the Lez River in South-West Ariège, between the localities of Moulis (42° 57′ 43″N; 1° 05′ 30″E) and Le Pont (42° 52′ 32″N 0° 57′ 19″E). A total of 5 females laid 56 eggs (clutch size = 11.20 ± 3.56 eggs) between the 12/7/2014 and the 27/7/2014. Eggs were collected within 12 hours post laying and individually marked for (1) identification purposes with a pencil using a letter (coding for clutch of origin) and a number (egg number within each clutch) and (2) positioning purposes^13^ (eggs were kept throughout the experiment in the position they were originally found). We used a split-clutch design to ensure an even repartition of eggs from each clutch in each treatment (hereafter called “group” and “single”; Pearson Chi-square = 0.15; df = 4; P = 0.99). Because egg mass influences both embryo metabolism and hatching phenotype in water snakes^22^, eggs were ranked within each clutch from heaviest to lightest and evenly regrouped into two half-clutches to ensure similar average egg mass within each treatment: egg mass averaged 4.89 ± 0.28 g in group versus 4.92 ± 0.28 g in single eggs (Anova with treatment and mother as factors, egg mass as variable; interaction term F_4, 46_ = 0.29; P = 0.89; effect of treatment F_1, 46_ = 0.14; P = 0.71). For each clutch, one half-clutch was incubated with eggs clustered (group treatment; N = 28 eggs; average half-clutch size = 5.60 ± 1.95 eggs), and the other with eggs separated into individual goblets (single treatment; N = 28 eggs).

### Egg mass, embryo heart rate and hatchling body size

Eggs were measured in mass to the nearest 0.01 g using a digital scale within 12 hours of oviposition and then every 10 days until hatching. We measured embryo heart rates using the Buddy^®^ digital egg monitor (MK2, Avitronics) under the standardised protocol described for eggs^22^,^44^. The Buddy^®^ system works by shining an infrared beam onto the surface of the egg, detecting minute distortions caused by embryonic heart beats. The Buddy^®^ monitor was left inside the incubator at all times to prevent temperature variation during heart rate readings. Each egg was gently placed onto the sensor pad for heart rate reading (a stable reading was obtained after approximately 30 seconds) and then returned to its clutch. Embryo heart rates were measured at incubation day 10, 20 and 30, and then every two days until hatching. All eggs were individually placed into small jewellery bags (5 × 4 cm, made of fine mesh material) a few days prior to hatching. This ensured juvenile snakes could be matched to their egg shell when multiple births occurred at the same time, while maintaining physical contact amongst group eggs. Hatchlings were measured in body mass (±0.01 g) and snout**-**vent length (±0.1 mm) within 12 hours of emergence.

### Hatchling social behaviour

All young snakes were kept in individual opaque containers (20 × 20 × 10 cm with water and shelter) for 2 weeks prior to testing (i.e. to allow all unabsorbed yolk to be assimilated) as to minimise sensory contacts amongst siblings. For the purpose of the analysis we needed equal numbers of snakes from each clutch and each treatment (see below). We randomly selected 5 snakes from the group treatment and 5 snakes from the single treatment from 4 of the 5 clutches (one clutch had too few snakes to be included in the behavioural tests). At 16h00 on testing day, the two groups of 5 snakes were each placed in an open top plastic box (60 × 40 × 40 cm), where direct physical contact between siblings (within treatment group) occurred for the first time. A digital video camera was fitted above the testing arenas and recorded the snake’s activities for 2.25 hours. The video was then edited on a computer and the data analysed. The first 15 minutes (acclimation time) were discarded. Every two minutes for a two-hour period we calculated the area occupied by the 5 snakes on a snapshot of the video within each box using open source software ImageJ (see Fig. 3). Every 15 minutes we measured the distance covered by each snake during one minute bouts using Photofiltre 7 with the plugin “mesures”. Behavioural traits were also recorded: moving *versus* immobile, and making contact with siblings.

All experimental protocols were approved by the Préfecture de l’Ariège, which provided capture, breeding, experimentation, release and ethics permits (Arrété #2012**-**11). All experiments were carried out in accordance with the approved guidelines. All females were returned to their exact site of capture within two weeks of egg**-**laying. Once tests were completed, young snakes were given their first meal (small dead minnows ranging from 0.5 g to 1 g; supplied by Armorvif^®^) prior to being released at the maternal capture site.

### Data analysis

Assumptions for normality of the data and equality of variances were tested on all variables (Lilliefors and Levene’s tests). Means ± standard deviations are given unless otherwise stated.

## Additional Information

**How to cite this article**: Aubret, F. *et al*. Only child syndrome in snakes: Eggs incubated alone produce asocial individuals. *Sci. Rep.* **6**, 35752; doi: 10.1038/srep35752 (2016).

## Figures and Tables

**Table 1:** Statistical results of a repeated measure Anova with treatment (group *versus* single eggs) and clutch of origin (N = 5) as factors and four successive egg masses as the repeated measure over time.

	Dl; F	P
{1} Clutch of origin	4; 21.67	0.001
{2} Treatment	1; 0.16	0.69
{1} * {2}	4; 0.39	0.82
{3} Time	3; 115.50	0.001
{1} * {3}	12; 30.94	0.001
{1} * {2}	3; 0.015	0.99
{1} * {2} * {3}	12; 4.31	0.001

**Table 2:** Statistical results of a repeated measure Anova with treatment (group *versus* single eggs) and clutch of origin (N = 5) as factors and 7 successive embryonic heart rates as the repeated measure over time.

	Dl; F	P
{1} Clutch of origin	4; 9.22	0.001
{2} Treatment	1; 1.21	0.28
{1} * {2}	4; 1.37	0.26
{3} Time	6; 112.80	0.001
{1} * {3}	24; 4.27	0.001
{1} * {2}	6; 7.88	0.001
{1} * {2} * {3}	24; 1.75	0.001

**Table 3:** Two-way Anovas with treatment and clutch of origin as factors and relevant traits were performed.

Traits	Group eggs N = 28	Single eggs N = 28	df; F	P
Incubation time (days)	44.18 ± 0.22	44.62 ± 0.22	1, 46; 1.97	0.17
Unabsorbed yolk (g)	0.28 ± 0.05	0.43 ± 0.05	1, 46; 5.37	**0.025**
Body mass (g)	2.72 ± 0.09	2.56 ± 0.09	1, 46; 2.30	0.14
Snout-vent length (cm)	13.55 ± 0.18	13.45 ± 0.18	1, 46; 0.18	0.68
